# Ethnopharmacological evaluation and metabolic mechanism of Sanjie Xiaoliu Granule in breast cancer with depression

**DOI:** 10.1186/s12575-026-00334-4

**Published:** 2026-05-18

**Authors:** Zhuo Liu, Tianhao Tong, Renyi Yang, Puhua Zeng

**Affiliations:** 1 Hunan Provincial Hospital of Integrated Traditional Chinese and Western Medicine (The Affiliated Hospital of Hunan Academy of Traditional Chinese Medicine), Changsha, 410006 China; 2 College of Traditional Chinese Medicine, Changsha Medical University, Changsha, 410219 China; 3 Institute of Traditional Chinese Medicine Oncology, Hunan Academy of Traditional Chinese Medicine, Changsha, 410006 China

**Keywords:** Breast cancer, Depression, Traditional Chinese medicine, SJXLG, Bioinformatics

## Abstract

**Background:**

Sanjie Xiaoliu Granule (SJXLG), a traditional multi-herbal prescription widely used in Chinese medicine, has been historically applied to “disperse nodules, soothe the liver, and relieve depression.” It is clinically prescribed for patients with breast lumps, emotional stagnation, and cancer-related depression. However, the pharmacological basis underlying its dual anticancer and antidepressant effects remains poorly understood.This study aimed to evaluate the therapeutic efficacy and elucidate the molecular and metabolic mechanisms of SJXLG in breast cancer with depression (BCD) through integrated experimental and bioinformatics approaches.

In vivo and in vitro BCD models were established to assess tumor growth, behavioral performance, and inflammatory cytokine levels. Liquid chromatography–mass spectrometry (LC–MS) identified active constituents of SJXLG. Bioinformatics analyses were used to screen key genes and signaling pathways, followed by validation via qRT-PCR and Western blot. Untargeted metabolomics was applied to explore the metabolic alterations in tumor tissues after SJXLG intervention.

**Results:**

SJXLG significantly inhibited breast cancer cell proliferation, migration, and invasion, and improved depressive-like behaviors in tumor-bearing mice. The formula upregulated MAOA, LTF, PTGER3, and IGFBP6 expression and regulated multiple signaling pathways, including PPAR, AMPK, and PI3K-Akt/mTOR, which are involved in lipid metabolism, inflammation, and neurotransmitter synthesis. Untargeted metabolomics further revealed that SJXLG restored metabolic homeostasis by modulating lipid, amino acid, and energy metabolism, alleviating oxidative stress, and rebalancing neurotransmitter-related metabolites. These findings suggest that SJXLG exerts integrative effects through multi-target, multi-pathway regulation.

**Conclusions:**

SJXLG demonstrates a synergistic anticancer and antidepressant effect through the modulation of metabolic and signaling networks associated with inflammation, oxidative stress, and neurotransmission. These results provide experimental evidence for its ethnopharmacological application in managing body–mind comorbidities such as breast cancer with depression and highlight its potential as a safe and effective adjunct therapy.

## Highlights

Sanjie Xiaoliu Granule (SJXLG) exerts dual antitumor and antidepressant effects in breast cancer with depression.

SJXLG suppresses breast cancer cell proliferation and migration while ameliorating depressive-like behaviors in tumor-bearing mice.

Integrated bioinformatics and metabolomics reveal coordinated regulation of lipid metabolism, inflammatory signaling, and neurotransmitter-related pathways by SJXLG.

Nineteen hub targets were identified, with MAOA, LTF, PTGER3, and IGFBP6 emerging as key protective genes associated with favorable prognosis.

Correlation analysis links altered metabolites with key molecular targets, supporting a molecular–metabolic regulatory axis underlying SJXLG efficacy.

## Introduction

Breast cancer is the most commonly diagnosed malignancy among women worldwide, with both incidence and mortality continuing to rise in recent years, posing a substantial public health burden [1]. In 2024, the global incidence of breast cancer is projected to reach 2.309 million new cases, representing an approximately 2.2% increase compared with 2020, thereby maintaining its position as the leading cancer among women [2].

Owing to its high incidence, prolonged treatment course, and substantial physical and psychosocial burden, breast cancer is frequently accompanied by depression, which represents one of the most prevalent comorbidities in affected patients [3, 4]. Epidemiological evidence indicates that approximately 20–30% of patients develop depressive symptoms during diagnosis and treatment [5]. Depression not only compromises quality of life and treatment adherence but also contributes to tumor progression through dysregulation of immune-inflammatory responses, neuroendocrine signaling, and neurotransmitter metabolism [6, 7]. Consequently, breast cancer with depression (BCD) is increasingly recognized as a complex mind–body comorbidity involving chronic inflammation, neurotransmitter imbalance, hypothalamic–pituitary–adrenal axis dysfunction, oxidative stress, and mitochondrial impairment [8, 9]. Current clinical management typically relies on the parallel use of anticancer therapies and antidepressants,however, chemotherapy, radiotherapy, and targeted agents are associated with substantial toxicity, while antidepressants may introduce drug–drug interactions and unintended effects on the tumor immune microenvironment [10]. These limitations underscore the urgent need for integrative therapeutic strategies capable of simultaneously addressing tumor progression and emotional disturbances in BCD. Sanjie Xiaoliu Granule (SJXLG) is a Traditional Chinese Medicine (TCM) formulation (Preparation number: Xiangyao Z20200306000) that has been clinically prescribed for breast disorders accompanied by emotional disturbances. SJXLG is administered orally at a dose of 10 g per package, three times daily, and is derived from classical TCM prescriptions. The formula consists of *Codonopsis pilosula* (Franch.) Nannf., *Astragalus mongholicus* Bunge, *Ganoderma lucidum* (Curtis) P. Karst., *Ostrea gigas* Thunberg, *Fritillaria thunbergii* Miq., *Prunella vulgaris* L., *Angelica sinensis* (Oliv.) Diels, *Curcuma aromatica* Salisb., *Vitex trifolia* L., *Vaccaria hispanica* (Mill.) Rauschert, and *Taraxacum mongolicum* Hand.-Mazz, in defined proportions. All botanical names were verified through the Medicinal Plant Name Services (MPNS, http://mpns.kew.org). According to traditional medical theory, SJXLG has been prescribed to “invigorate Qi and strengthen the body, soften hardness and dissipate nodules, clear heat and detoxify, regulate Qi and resolve stasis, and calm the mind to relieve depression.” These traditional indications are supported by classical medical texts such as *Yi Zong Jin Jian*, *Wai Ke Zheng Zong*, and *Ben Cao Gang Mu*, which document the use of key components (e.g., *Prunella vulgaris*, *Curcuma aromatica*, and *Ostrea gigas*) in conditions characterized by breast masses, Qi stagnation, and emotional constraint [11]. Recent clinical and experimental studies have provided modern evidence supporting the therapeutic value of SJXLG. Clinical investigations reported that SJXLG, when combined with chemotherapy, improved TCM symptom scores, reduced tumor marker levels, enhanced immune function, alleviated depressive symptoms, and mitigated chemotherapy-induced toxicity in patients with advanced breast cancer [12]. Additional clinical observations suggested benefits in patients with triple-negative breast cancer and chest wall metastasis, including pain relief and delayed disease progression [13]. Experimental studies further demonstrated that SJXLG inhibits epithelial–mesenchymal transition via suppression of the TGF-β1/Smads pathway and downregulates RECQL4 expression, implicating its role in regulating tumor proliferation and DNA repair [14]. Nevertheless, despite these findings, the integrated molecular mechanisms underlying the dual antitumor and antidepressant effects of SJXLG in BCD remain incompletely understood.

Therefore, the present study aimed to systematically elucidate the multi-target mechanisms of SJXLG in breast cancer with comorbid depression by integrating network pharmacology, untargeted metabolomics, behavioral assessments, and molecular validation using animal models and cellular experiments. This work seeks to bridge the ethnopharmacological foundations of SJXLG with modern mechanistic evidence, providing a scientific basis for its application in the management of complex tumor–psychiatric comorbidities.

## Materials and methods

### Chemicals and reagents

Methanol (Thermo Fisher Scientific, A452-4, Fisher), acetonitrile (Thermo Fisher Scientific, A998-4, Fisher), formic acid (Thermo Fisher Scientific, A117-50, Fisher), and ultrapure water were used. MEM medium, RPMI-1640 medium, fetal bovine serum (FBS), and penicillin–streptomycin solution were obtained from Wuhan Procell Life Science & Technology Co., Ltd. (cat. nos. PM150410, PM150110, 164220, PB180122). Capecitabine (Jiangsu Hengrui Medicine Co., Ltd., H20133365) and Fluoxetine (Shandong Linuo Pharmaceutical Co., Ltd., H20123161).CCK-8 Cell Viability Assay Kit, EdU Cell Proliferation Assay Kit, JC-1 dye, and Mitored dye were purchased from Beyotime Biotechnology (cat. nos. C0037, C0085S, C2003S, C1032). Antibodies included: E-cadherin (Wuhan Sanying, 20874–1-AP), N-cadherin (Affinity, AFRM80077), Ki-67 (Proteintech, 28074–1-AP), MAOA (Proteintech, 10539–1-AP), LTF (Proteintech, 31267–1-AP), PTGER3 (Proteintech, 14357–1-AP), β-Actin (Proteintech, 66009–1-Ig), and IGFBP6 (Abcam, ab242524). Cytokine ELISA kits for CA15-3 (ML058464), IL-1β (ML098416), IL-18 (ML1066842), IL-6 (MI098430) were purchased from mlbio (Shanghai, China); 5-HTP (ZC56074), 5-HT (ZC37715), and glutamate (ZC38187) ELISA kits from ZCIBIO (Shanghai, China). HRP-conjugated goat anti-rabbit IgG (H + L), CoraLite488-conjugated goat anti-rabbit IgG (H + L), and CoraLite594-conjugated goat anti-rabbit IgG (H + L) were purchased from Proteintech Group (cat. nos. SA00001-2, SA00013-2, SA00013-4).

### Preparation of SJXLG aqueous extract

Herbal materials of SJXLG were provided by the Pharmacy of Hunan Provincial Hospital of Integrated Traditional Chinese and Western Medicine (Table 1). Each medicinal material was authenticated by the pharmacognosist Yun Qiu, and its botanical name was verified through authoritative databases, including the *Medicinal Plant Name Services* (http://mpns.kew.org) and *The Plant List* (www.theplantlist.org), to ensure the accuracy and consistency of identification. The formula consists of Codonopsis pilosula (Franch.) Nannf., Astragalus mongholicus Bunge, Ganoderma lucidum (Curtis) P. Karst., Ostrea gigas Thunberg, Fritillaria thunbergii Miq., Prunella vulgaris L., Angelica sinensis (Oliv.) Diels, Curcuma aromatica Salisb., Vitex trifolia L., Vaccaria hispanica (Mill.) Rauschert, and Taraxacum mongolicum Hand.-Mazz. The herbs were soaked in 4 volumes of distilled water for 30 min, decocted for 30 min, and filtered. The residue was decocted again under the same conditions. Both extracts were combined and concentrated under reduced pressure to 2.0 g/mL, then stored at 4°C.

**Table 1:** The compositions of SJXLG

Materials	TCM materials name	Latin name	Part used	Location
Codonopsis Radix	Dangshen	*Codonopsis pilosula* (Franch.) Nannf	Root	Gansu
Astragali Radix	Huangqi	*Astragalus membranaceus* (Fisch.) Bge	Root	Neimeng
Fructus Ligustri Lucidi	Nvzhenzi	*Ligustrum lucidum* Ait	Dry fruit	Hunan
Epimedii Folium	Xianlingpi (Yinyanghuo)	*Epimedium brevicornu* Maxim	Dry leaf	Shanxi
Prunellae Spica	Xiakucao	*Prunella vulgaris* L	Spike	Henan
Rhapontici Radix	Loulu	*Echinops latifolius* Tausch	Root	Hebei
Pinelliae Rhizoma	Fabanxia	*Pinellia ternata* (Thunb.) Breit	Rhizome	Henan
Curcumae Rhizoma	Ezhu	*Curcuma phaeocaulis* Valeton	Rhizome	Sichuan
Scutellariae Barbatae Herba	Shijianchuan	*Scutellaria barbata* D.Don	Whole herb	Anhui
Selaginellae Herba	Shishangbai	*Selaginella tamariscina* (Beauv.) Spring	Whole herb	Shaanxi
Curcumae Radix	Yujin	*Curcuma wenyujin* Y.H.Chen & C.Ling	Rhizome	Sichuan
Albiziae Cortex	Hehuanpi	*Albizia julibrissin* Durazz	Bark	Jiangsu

### Preparation of SJXLG and drug-containing serum

Twenty Sprague–Dawley rats were randomly divided into SJXLG and control groups (*n* = 10/group). The SJXLG group was administered the clinical equivalent dose (3.9 g/kg) by gavage for 7 consecutive days, while the control group received distilled water. One hour after the last administration, rats were anesthetized with sodium pentobarbital (3%, 30 mg/kg), and blood was collected via the abdominal aorta. Serum was separated by centrifugation (3000 rpm, 15 min, 4 °C), inactivated, and stored at –80 °C.

### Collection of active components in SJXLG

A 100 mg sample of SJXLG was ground uniformly in liquid nitrogen and then mixed with 1 mL of water containing 4 μg/mL of a composite internal standard. The mixture was vortexed for 1 min with the addition of stainless-steel beads. Samples were frozen at −40 °C for 2 min and subsequently subjected to grinding in a tissue grinder (60 Hz, 2 min), followed by ultrasonic extraction in an ice-water bath for 60 min. The extract was centrifuged at 12,000 rpm for 10 min at 4 °C, and the supernatant was diluted fivefold with water containing the internal standard. Finally, 200 μL of the supernatant was transferred into LC–MS vials equipped with inserts for analysis.

Analysis was performed using an ACQUITY UPLC I-Class HF ultra-performance liquid chromatography system coupled to a QE high-resolution mass spectrometer. Chromatographic separation was achieved using an ACQUITY UPLC HSS T3 column (100 mm × 2.1 mm, 1.8 μm) at 45 °C. Mobile phase A consisted of water with 0.1% formic acid, and mobile phase B was acetonitrile, with a flow rate of 0.35 mL/min. The injection volume was 2 μL. Mass spectrometry was conducted using a heated electrospray ionization (HESI) source in both positive and negative ion modes.

LC/TOF data were recorded using Analyst TF 1.6.2 software. Prior to pattern recognition, raw data were preprocessed using the metabolomics software XCMS v4.5.1 for baseline correction, peak detection, integration, retention time correction, peak alignment, and normalization. Compound identification was based on accurate mass, secondary fragment ions, and isotopic distribution, with qualitative confirmation using a TCM database. Further interpretation was conducted using both online resources and proprietary databases.

Chemical components were further screened using the TCM Systems Pharmacology (TCMSP) database, with oral bioavailability (OB) and drug-likeness (DL) thresholds as selection criteria. Specifically, molecules with OB ≥ 30% and DL ≥ 0.18 were considered active. Additional components were identified based on documented bioactivity, high abundance, or detection in human plasma.

### Cell culture

The human breast cancer cell line MCF-7 and the murine breast cancer cell line 4T1 were obtained from the Cell Bank of the Chinese Academy of Sciences (Shanghai, China). MCF-7 cells were cultured in DMEM high-glucose medium supplemented with 10% FBS and 1% penicillin–streptomycin, while 4T1 cells were maintained in RPMI-1640 medium containing 10% Gibco FBS and 1% penicillin–streptomycin. All cells were incubated at 37 °C in a humidified atmosphere with 5% CO_2_₂Cells in the logarithmic growth phase were used for subsequent experiments. To simulate a stress environment, MCF-7 cells were co-incubated with 200 μM corticosterone (CORT) for 15 min to establish the BCD cell model [15].

### CCK-8 assay

MCF-7 cells in the logarithmic growth phase were seeded into 96-well plates at a density of 5,000 cells per well in 100 μL of complete medium. To exclude the potential effect of blank serum on cell viability, cells were divided into control, model, 10% blank serum, and 10% SJXLG groups, and treated for 24 h. In addition, blank serum control, blank serum model, and different concentrations of SJXLG drug-containing serum (5%, 10%, 15%, and 20%) were applied for 24 h and 48 h, respectively. Each group included three replicate wells. After treatment, 10 μL of CCK-8 solution was added to each well, followed by incubation at 37 °C for 60 min. Absorbance was measured at 450 nm using a multifunctional microplate reader, and cell viability was calculated accordingly.

### EdU incorporation assay

Cells were plated in 96-well plates (5 × 10^3^ cells/well) and treated as above. EdU solution (10 µM) was added, incubated for 2 h, followed by fixation with 4% paraformaldehyde. Apollo® staining and Hoechst counterstaining were performed. Positive nuclei were imaged with a fluorescence microscope (Leica).

### Colony formation assay

MCF-7 cells were seeded in 6-well plates at a density of 500 cells per well. After modeling, cells in the control and model groups were treated with 10% blank serum, whereas cells in the experimental groups were treated with SJXLG at concentrations of 5%, 10%, and 20%. Culture medium was refreshed every 3 days. After 11 days, cells were fixed and stained with crystal violet. Plates were air-dried, imaged, and colonies were counted.

### Wound healing assay

MCF-7 cells were seeded in 6-well plates at 2 × 10^5^ cells per well. After modeling, cells in the control and model groups were treated with 10% blank serum, while experimental groups were treated with SJXLG at concentrations of 5%, 10%, and 20%. Once cells reached confluence, a straight scratch was made in each well using a 200 μL pipette tip, followed by three washes with PBS to remove detached cells. Images of the wound area were captured at 0, 24, and 48 h under a microscope. Wound closure was analyzed using Image Pro Plus 6.0 software, and the wound migration rate was calculated as follows:Scratch Migration Rate = [(Scratch Width Immediately After Scratching—Scratch Width at the Specified Time Point After Scratching)/Scratch Width Immediately After Scratching] × 100%

### Transwell cell invasion assay

MCF-7 cells were seeded in the upper chamber of Matrigel-coated Transwell inserts at a density of 2 × 10^4^ cells per well in 200 μL serum-free medium. The lower chamber was filled with 750 μL culture medium. After modeling, cells in the control and model groups were treated with 10% blank serum, whereas cells in the experimental groups were treated with SJXLG drug-containing serum at concentrations of 5%, 10%, and 20%. Following 24 h of incubation, non-invading cells on the upper surface of the insert were gently removed with a cotton swab. Inserts were washed with PBS, fixed with paraformaldehyde, and stained with crystal violet. Invaded cells on the lower surface were imaged under a microscope and quantified using ImageJ software.

### Mito-tracker green staining for mitochondrial morphology

Mito-Tracker Green was used to visualize mitochondrial morphology. MCF-7 cells were seeded in 24-well plates at 1 × 10^5^ cells per well and cultured overnight. After 24 h of drug treatment, cells were washed three times with PBS and incubated with 0.5 μM Mito-Tracker Green for 15 min. Following three additional PBS washes, mitochondrial morphology was observed and imaged using a laser confocal microscope.

### JC-1 staining assay

JC-1 dye was used to assess mitochondrial membrane potential (MMP) as an indicator of mitochondrial function. After 24 h of drug treatment, MCF-7 cells were washed twice with PBS and incubated with 1 mL JC-1 working solution at 37 °C for 20 min. Cells were then washed twice with JC-1 buffer. Healthy cells exhibited red fluorescence (590 nm) due to JC-1 aggregation, while apoptotic or necrotic cells displayed green fluorescence (530 nm). Fluorescence intensity ratios (red/green) were calculated under an inverted fluorescence microscope to evaluate mitochondrial function.

### ELISA for cytokine quantification in conditioned media

After 18 h of intervention, conditioned media were collected and centrifuged at 3000 rpm for 15 min at 4 °C to remove cellular debris. The supernatants were analyzed for IL-1β, IL-18, and IL-6 concentrations using commercially available ELISA kits according to the manufacturer’s instructions.

### Transmission electron microscopy (TEM)

After 24 h of treatment, cells were fixed with 2.5% glutaraldehyde at room temperature. Following a 5-min initial fixation, cells were collected, centrifuged, and resuspended in fresh TEM fixative, then stored at 4 °C for 30 min. Samples were subsequently dehydrated, embedded, and sectioned. Ultrastructural morphology of mitochondria was observed using a HITACHI HT7800 transmission electron microscope.

### Bioinformatics analysis

The GSE15852 dataset was retrieved from the GEO database (https://www.ncbi.nlm.nih.gov/geo/) [16]. Differential expression analysis was performed using the limma package in R, and genes with *P* < 0.05 and |log2(FC)|> 1 were considered differentially expressed. Genes associated with major depressive disorder (MDD) were obtained by querying the GeneCards database using the keyword “Major Depressive Disorder” [17].

For drug target prediction, the chemical structures of 24 active compounds identified in Sect. 2.4 were obtained from PubChem (https://pubchem.ncbi.nlm.nih.gov/) in SMILES or 3D SDF format. Potential targets were predicted using PharmMapper (http://www.lilab-ecust.cn/pharmmapper/), SwissTargetPrediction (http://www.swisstargetprediction.ch/), and TargetNet (http://targetnet.scbdd.com/) [18]. Redundant targets were removed, and the remaining targets were converted to gene symbols using the UniProt database (https://www.uniprot.org/). The intersection of SJXLG targets and differentially expressed genes was analyzed using R (ggplot2) to identify potential key genes involved in SJXLG intervention in breast cancer-associated depression [18].

For TCGA data analysis, RNA-seq data from the TCGA-BRCA project were downloaded from the TCGA database (https://portal.gdc.cancer.gov). Gene expression differences were analyzed in R [19]. For normally distributed data, unpaired Student’s *t*-tests were applied; for non-normal data, rank-sum tests were used. *P* < 0.05 was considered statistically significant.

Protein–protein interaction (PPI) networks were constructed using the STRING database [20]. GO and KEGG pathway enrichment analyses were performed with R packages clusterProfiler and org.Hs.eg.db to explore potential mechanisms of SJXLG in breast cancer. Nineteen genes at the intersection of differentially expressed genes and SJXLG targets were further analyzed. Lasso regression was applied to select genes with high prognostic relevance, combined with cross-validation. Analyses were performed using the glmnet package in R (v4.2.1) to determine the optimal λ, maximum likelihood, or C-index, and results were visualized. Univariate Cox regression was used to evaluate the association between individual genes and patient survival. The intersection of prognostic genes identified by Lasso regression and univariate Cox regression was determined using ggplot2 [21].

Finally, multivariate Cox regression was performed to identify SJXLG target genes associated with breast cancer prognosis in depression. Kaplan–Meier survival curves were plotted to compare high- and low-risk groups using the survminer and ggplot2 packages. The predictive performance of key gene-based risk scores for 1-, 3-, and 5-year survival was evaluated using the timeROC package in R.

### Animal experiments and treatments

C57BL/6 mice were purchased from Hunan Slack Jingda Laboratory Animal Co., Ltd. (SCXK: 2019–0004) and housed at the Animal Experiment Center of Hunan University of Chinese Medicine under a 12 h light/dark cycle with ad libitum access to food and water.

Animal housing and management were performed in accordance with the “Guide for the Humane Handling of Laboratory Animals.” After one week of adaptive feeding, 4T1 breast cancer cells were injected into the right fourth mammary fat pad of mice to establish a breast cancer model. Seven days after tumor cell injection, tumor-bearing mice were randomly assigned to six groups (n = 5 per group, total 30 mice): control group, model group, and SJXLG groups receiving low (1.3 g·kg^−1^, SJXLG-L), medium (1.95 g·kg^−1^, SJXLG-M), and high (3.9 g·kg^−1^, SJXLG-H) doses of SJXLG by oral gavage; the positive drug group was treated with capecitabine (60 mg·kg^−1^) and fluoxetine (19.5 mg·kg^−1^); the model group received an equal volume of distilled water. When tumor volumes reached approximately 80 mm^3^, except for the model group, mice in the remaining groups were subcutaneously injected in the dorsal region with CORT suspension (30 mg·kg^−1^) for 14 consecutive days to establish a mouse model of breast cancer with comorbid depression and to administer treatments [15].

### Behavioral assessments

#### Open field test (OFT)

Each mouse was placed in a 50 × 50 × 40 cm test chamber and allowed to acclimate for 30 s. Locomotor activity was recorded for 3 min, including total distance traveled, number of entries into the central area, and time spent in the central zone.

#### Tail suspension test (TST)

Following 24 h food deprivation, mice were suspended by the tail 1–2 cm from the tip, 60 cm above the floor. Immobility time was recorded over 3 min.

#### Sucrose preference test (SPT)

Mice were first adapted to 1% sucrose solution for 3 days, with both sucrose and water bottles provided in the cage (positions alternated daily, with food available ad libitum). After adaptation, mice underwent 8–12 h of water deprivation (without food restriction) and were then given access to sucrose solution and water for 1 h. Fluid consumption was measured, and sucrose preference was calculated as: [sucrose intake/(sucrose intake + water intake)] × 100%.

#### Forced swim test (FST)

Mice were placed in a transparent cylinder (water depth 15–20 cm, 23–25 °C) for 6 min. Immobility during the last 4 min was recorded to assess depressive-like behavior.

### ELISA assays

After behavioral testing, mice were anesthetized with intraperitoneal injection of 3% pentobarbital sodium (30 mg·kg^−1^), and serum samples were collected. Concentrations of 5-HTP, 5-HT, Glu, IL-1β, IL-18, and IL-6 were quantified using ELISA kits according to the manufacturer’s instructions.

### Body weight and tumor volume measurement

Body weight was recorded every 3 days during treatment. After 14 days of intervention, mice were humanely euthanized, and tumor volumes were measured. Tumor volume (V) was calculated using the formula: V = (a × b^2^)/2,where *a* is tumor length and *b* is tumor width.

### Hematoxylin & Eosin (H&E) staining

Tumor tissues were fixed, embedded, and sectioned at ~ 4.5 μm. After deparaffinization and rehydration, sections were stained with hematoxylin for 3 min, rinsed, and counterstained with eosin for 5 min. Slides were mounted and imaged under a light microscope.

### Immunohistochemistry (IHC)

After deparaffinization and antigen retrieval, endogenous peroxidase activity was blocked. Sections were incubated overnight with primary antibodies against E-cadherin (1:5000) and N-cadherin (1:50), followed by HRP-conjugated secondary antibody for 50 min. DAB was used for visualization, and ImageJ software quantified the integrated optical density (IOD) or positive cell percentage.

### Immunofluorescence (IF) staining

Ki-67 expression was assessed by IF. After deparaffinization, hydration, and H_2_O_2_ treatment, sections were incubated overnight with Ki-67 antibody (1:200), followed by CoraLite 488-conjugated secondary antibody for 1 h and DAPI counterstaining. Fluorescence microscopy was used to capture images and determine the percentage of positive cells.

### Quantitative real-time PCR (qRT-PCR)

Tumor tissues stored at –80 °C were sampled and ground in liquid nitrogen, followed by the addition of pre-cooled lysis buffer at a ratio of 50 mg tissue per 1 mL buffer. Total RNA was extracted using an ultrapure RNA extraction kit, and RNA concentration and purity were determined by ultraviolet spectrophotometry, with an A260/A280 ratio between 1.9 and 2.1 considered acceptable. First-strand cDNA was synthesized from RNA using a reverse transcription kit under the following conditions: incubation at 45 °C for 5 min, followed by incubation at 50 °C for 15 min after reagent addition. Real-time PCR was performed using the SYBR Green I fluorescence method in a two-step protocol (initial denaturation at 95 °C for 1 min; 35–45 cycles of 95 °C for 20 s and 60 °C for 1 min). Glyceraldehyde-3-phosphate dehydrogenase (GAPDH) was used as the internal control, and the relative expression of target genes was calculated using the 2-△△Ct method. The primers used in this study were designed by Sangon Biotech Co., Ltd. (Shanghai, China), detailed in Table 2.

**Table 2:** Primer design for MAOA, LTF, PTGER3, IGFBP6, and GAPDH

Gene	Forward primer sequence	Reverse primer sequence	Product length (bp)
MAOA	GACACGCTCAGGAATGGGACAAG	ACAGGAACCACAGGGCAGATACC	147
LTF	CCCAAACCATGCTGTAGTGTCTC	ACACCTCTGTCCATTTCTCTCCCAA	100
PTGER3	TGTTGGTCGCCGCTATTGATAA	TTCAGCGAAGCCAGGCGAA	150
IGFBP6	AGGAATCCAGGCACCTCTACCA	AGCACTGAGTCCAGATGTCTACGG	200
GAPDH	GCACCGTCAAGGCTGAGAAC	TGGTGAAGACGACAGTGGA	250

### Western blotting (WB)

Total protein was extracted from tumor tissues using RIPA buffer with PMSF. Homogenates were centrifuged on ice, and supernatants were collected. Protein concentration was measured by BCA assay. Fifty micrograms of protein were separated on 10% SDS-PAGE gels and transferred to PVDF membranes (0.45 μm). Membranes were blocked with 5% non-fat milk in TBST at 37 °C for 1 h, incubated overnight at 4 °C with primary antibodies against MAOA, LTF, PTGER3, IGFBP6, and β-actin (1:1000). After washing, HRP-conjugated secondary antibody (1:5000) was incubated at room temperature for 1 h, followed by visualization using a chemiluminescence system. Band intensity was quantified using ImageJ, with β-actin as the internal reference.

### Untargeted metabolomics analysis

Untargeted metabolomic profiling was performed using a UHPLC–Q Exactive Orbitrap system (Thermo Fisher Scientific, USA) equipped with a Hypersil GOLD C18 column (2.1 × 100 mm, 1.9 μm). Samples were analyzed in both positive and negative ion modes under a 0.3 mL/min gradient elution. Raw data were processed using Compound Discoverer 3.1 for peak alignment and normalization. Multivariate statistical analyses (PCA, OPLS-DA) were conducted with SIMCA 14.1, and significant metabolites were identified according to VIP > 1.0, FC > 1.5 or < 0.67, and *p* < 0.05. KEGG-based pathway enrichment was performed through MetaboAnalyst 5.0.

### Statistical analysis

Data are presented as mean ± standard deviation (SD). Statistical analysis was performed using SPSS 25.0. For normally distributed data with homogenous variance, one-way ANOVA was applied, followed by LSD post hoc tests; for unequal variance, Tamhane’s T2 test was used. Non-normally distributed data were analyzed using the Kruskal–Wallis test. *P* < 0.05 was considered statistically significant.

## Results

### Identification of compounds from SJXLG

**Fig. 1:**
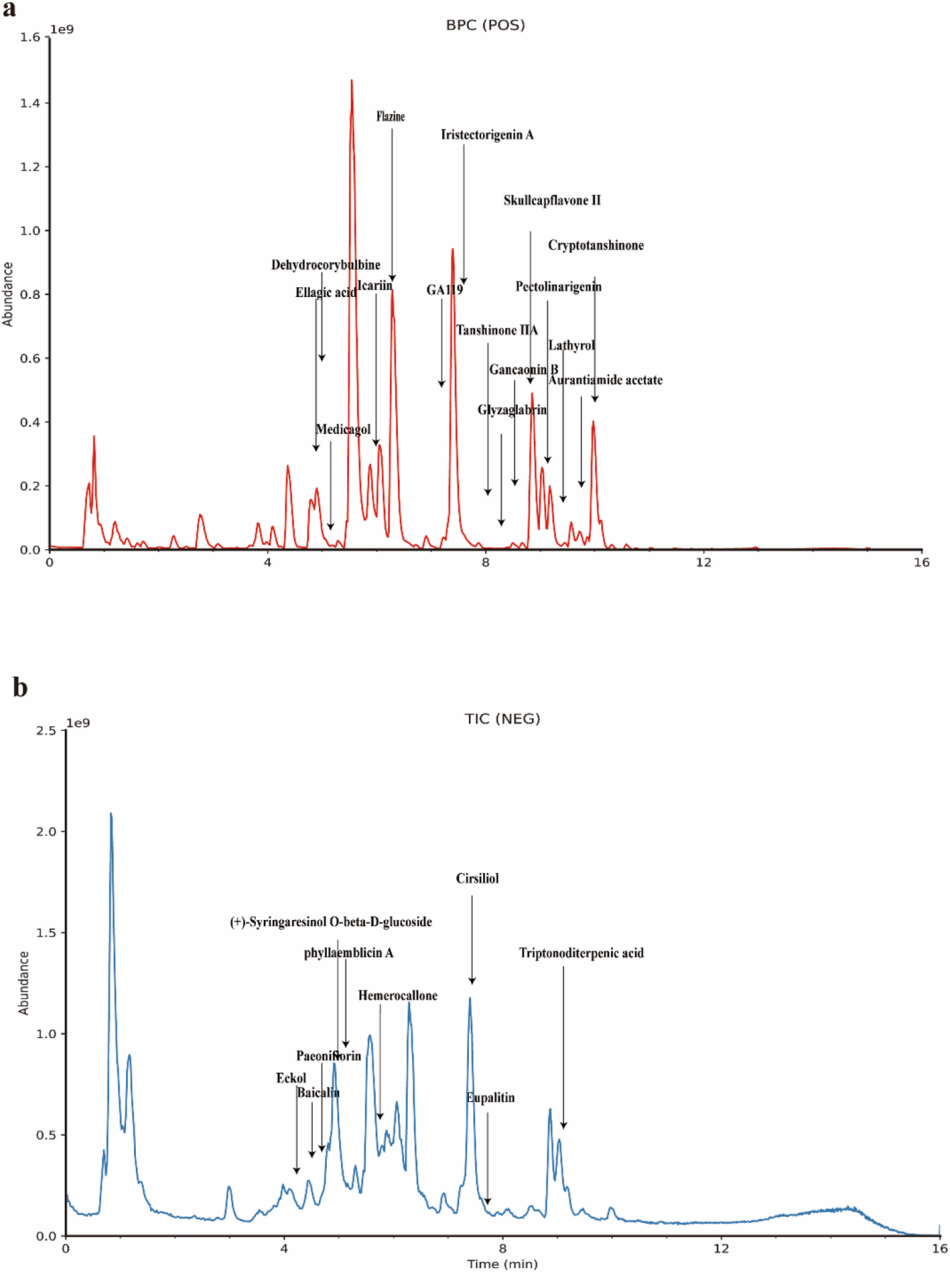
Base peak chromatograms (BPCs) of SJXLG. **a** Positive ion mode BPC of SJXLG. **b** Negative ion mode BPC of SJXLG

**Fig. 2:**
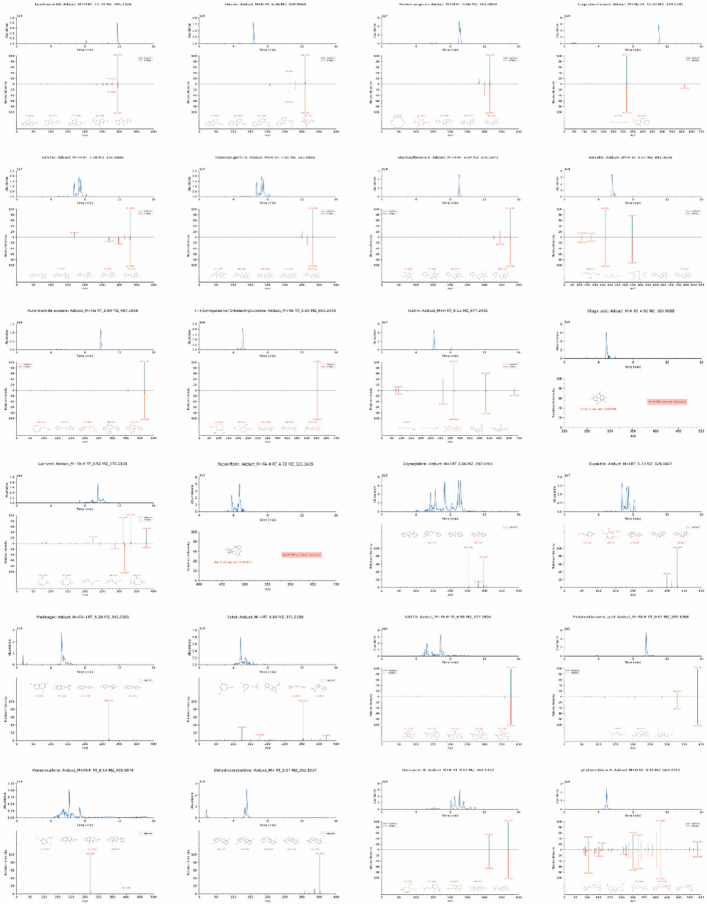
MS/MS spectra of 24 bioactive components identified in SJXLG

A total of 1,252 unique chemical constituents were identified in SJXLG. The comprehensive total ion chromatogram (TIC) (Fig. 1) revealed a broad spectrum of compounds. Based on rigorous screening criteria, and supported by extensive literature review, 24 active components were selected from 268 chemical constituents (Fig. 2). These included Tanshinone IIA, Flazine, Pectolinarigenin, Cryptotanshinone, Cirsiliol, Iristectorigenin A, Skullcapflavone II, Baicalin, Aurantiamide acetate, (+)-Syringaresinol O-β-D-glucoside, Icariin, Ellagic acid, Lathyrol, Paeoniflorin, Glyzaglabrin, Eupalitin, Medicagol, Eckol, GA119, Triptonoditerpenic acid, Hemerocallone, Dehydrocorybulbine, Gancaonin B, and Phyllaemblicin A (see Table 3). These bioactive constituents encompassed five isoflavonoids, one lignan, one flavonol, one polyphenol, three diterpenes, three phenylpropanoids, one peptide, and one monoterpene.

**Table 3:** Summary of mass spectrometry analysis of the 24 active compounds identified in SJXLG

Name	PubChem ID	Molecular Formula	Ion Mode	Retention Time (min)	Precursor Ion (m/z)	Fragment Ion(m/z)
Tanshinone IIA		C19H18O3	M + H	11.787	295.1323	249.12701, 277.1218, 295.13242, 296.13571
Flazine	5377686	C17H12N2O4	M + H	6.357	309.0858	263.08127, 281.0918, 309.08676, 309.168, 310.09015
Pectolinarigenin	5320438	C17H14O6	M + H	9.063	315.0855	282.05179, 285.03876, 300.06247, 315.08575, 316.08942
Cryptotanshinone	5320438	C17H14O6	M + H	9.063	315.0855	282.05179, 285.03876, 300.06247, 315.08575, 316.08942
Cirsiliol	160237	C17H14O7	M + H	7.302	331.08	169.01303, 316.0575, 331.08078
Iristectorigenin A	5488781	C17H14O7	M + H	7.497	331.0798	298.04694, 301.03366, 316.05728, 331.0806
Skullcapflavone II	124211	C19H18O8	M + H	9.043	375.1067	327.04913, 345.05978, 375.10684, 376.10999
Baicalin	64982	C21H18O11	2 M-H	5.569	891.1647	113.02451, 175.02483, 269.04562, 445.07791
Aurantiamide acetate	124319	C27H28N2O4	M + Na	9.836	467.1931	467.19418
(+)-Syringaresinol O-beta-D-glucoside	443024	C28H36O13	M + Na	5.08	603.2036	603.2052, 603.29651
Icariin	5318997	C33H40O15	M + H	6.109	677.243	71.04977, 85.02891, 313.0701, 369.13272, 531.18597, 677.24121
Ellagic acid	5281855	C14H6O8	M-H	4.919	300.9986	
Lathyrol	5281376	C20H30O4	M + FA-H	9.516	379.2076	87.04501, 208.33835, 223.17065, 305.16208, 311.22275, 333.20471, 334.20865, 379.20978
Paeoniflorin	425990	C23H28O11	M + FA-H	4.716	525.1624	
Glyzaglabrin	5317777	C16H10O6	M-H	8.959	297.0396	253.05058, 282.01663, 297.00439, 297.03967, 298.0459, 305.16211
Eupalitin	5748611	C17H14O7	M-H	6.726	329.0667	299.01974, 314.04349, 329.06686, 329.23331
Medicagol	5319322	C16H8O6	M + FA-H	5.28	341.0299	269.04553, 341.02863
Eckol	145937	C18H12O9	M-H	4.837	371.0363	125.02447, 177.019, 303.05118, 304.05533, 327.05069, 371.04022, 371.0874
GA119		C19H24O5	M + FA-H	6.876	377.1602	377.16043, 378.16364
Triptonoditerpenic acid	132263	C21H28O4	M + FA-H	9.569	389.1966	329.17569, 389.19699, 390.20029
Hemerocallone	5318001	C19H16O7	M + FA-H	6.136	401.0874	269.0455, 401.0885
Dehydrocorybulbine	5316439	C21H22NO4 +	M +	5.571	352.1531	308.12769, 336.12253, 337.13025, 352.15393, 353.15656
Gancaonin B	5317479	C21H20O6	M + H	9.103	369.1331	313.0701, 369.13272
phyllaemblicin A		C27H34O14	M + H	4.944	583.1999	349.1064, 355.1157, 367.1169, 385.1284, 403.1383, 421.1489, 445.1470, 547.1777, 583.2052, 583.3170

### SJXLG suppresses proliferation, migration, and invasion of breast cancer cells under chronic stress conditions

**Fig. 3:**
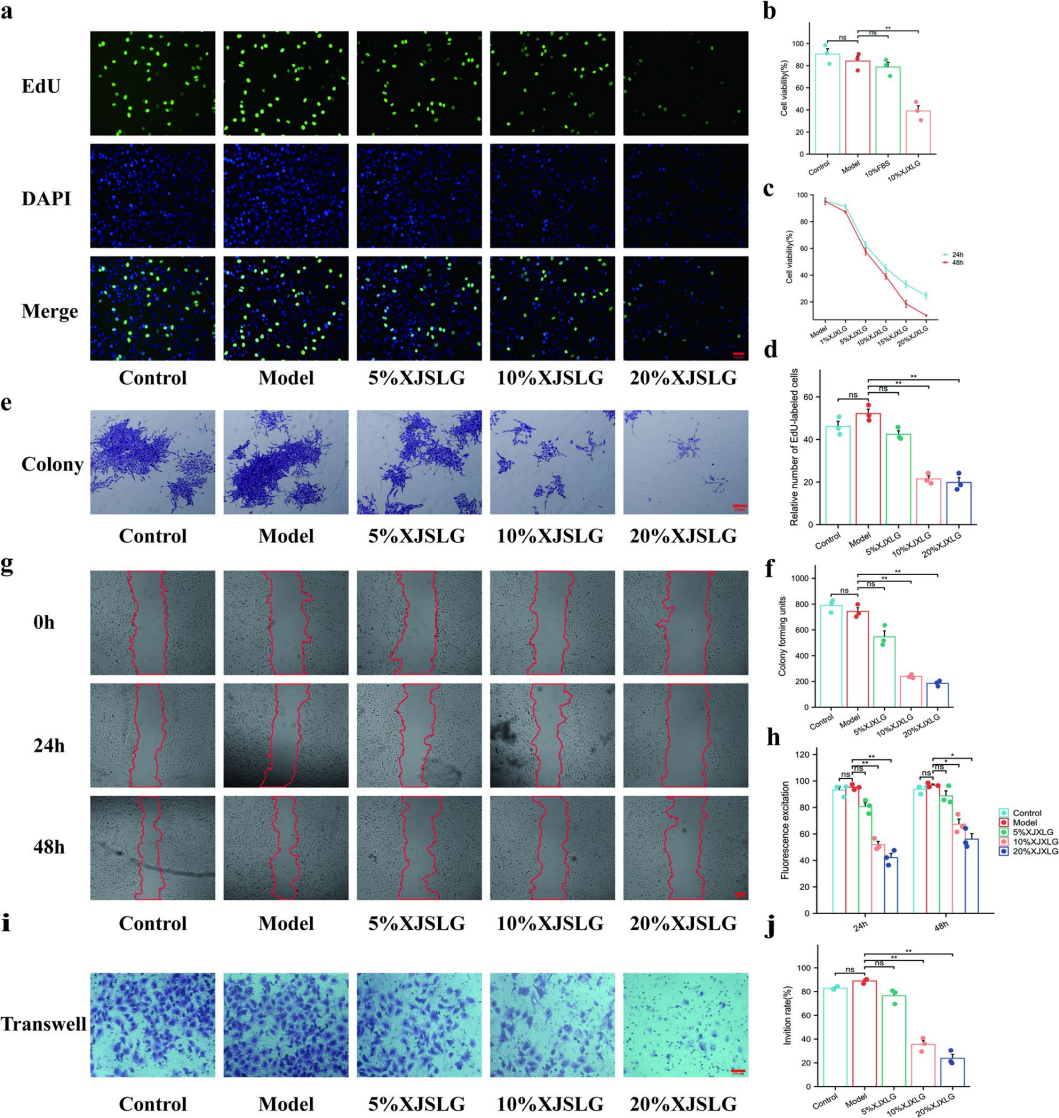
SJXLG inhibited the proliferation, invasion, and metastasis of CORT-MCF-7 cells in vitro. **a** EdU proliferation assay showing the effects of SJXLG-containing serum (5%, 10%, 20%) on CORT-MCF-7 cell proliferation. **b** Comparison of 10% SJXLG-containing serum and FBS on CORT-MCF-7 cells. **c** CCK-8 assay showing cell viability after treatment with SJXLG-containing serum (1%, 5%, 10%, 15%, 20%) for 24 h and 48 h. **d** Quantification of EdU-positive cells. **e** Colony formation assay of CORT-MCF-7 cells treated with SJXLG-containing serum (5%, 10%, 20%). **f** Quantification of colony formation. **g** Wound healing assay of CORT-MCF-7 cells treated with SJXLG-containing serum (5%, 10%, 20%). **h** Quantitative analysis of wound closure. **i** Quantification of invaded cells. **j** Transwell invasion assay showing the effects of SJXLG-containing serum (5%, 10%, 20%) on CORT-MCF-7 cell invasion. Data are presented as mean ± SD, *n* = 3. *P < 0.05, **P < 0.01 versus control

In this study, control, model, 10% blank serum, and 10% SJXLG were established and treated for 24 h. Additionally, blank serum control, blank serum model, and different concentrations of SJXLG (5%, 10%, 15%, and 20%) were tested at 24 h and 48 h. Results demonstrated that SJXLG inhibited the proliferation of MCF-7 breast cancer cells and attenuated the depressive breast cancer model phenotype. The inhibitory effect was both concentration- and time-dependent (*P* < 0.01, Fig. 3b,c). Based on these findings, subsequent experiments adopted a concentration of 10% SJXLG and a 24 h intervention period.

EdU incorporation assays revealed that SJXLG treatment significantly suppressed MCF-7 cell proliferation in the model group, particularly at 10% and 20% concentrations (*P* < 0.01, Fig. 3a,d).

Similarly, colony formation assays indicated a dose-dependent reduction in colony numbers with increasing SJXLG concentrations (5%, 10%, 20%), with significant suppression observed at 10% and 20% (*P* < 0.01, Fig. 3e,f). These results suggest that SJXLG inhibited proliferation of breast cancer cells in the depressive model in a dose-dependent manner, possibly by activating cell death pathways.

Scratch wound healing assays further demonstrated that SJXLG reduced cell migration at both 24 h and 48 h, with significant effects observed at 10% and 20% concentrations (*P* < 0.01, *P* < 0.05, Fig. 3g,h). Transwell invasion assays confirmed that SJXLG dose-dependently reduced the number of invasive cells, with significant effects at 10% and 20% (*P* < 0.01, Fig. 3i, j). These findings indicate that SJXLG effectively suppresses migration and invasion of breast cancer cells in the depressive model.

**Fig. 4:**
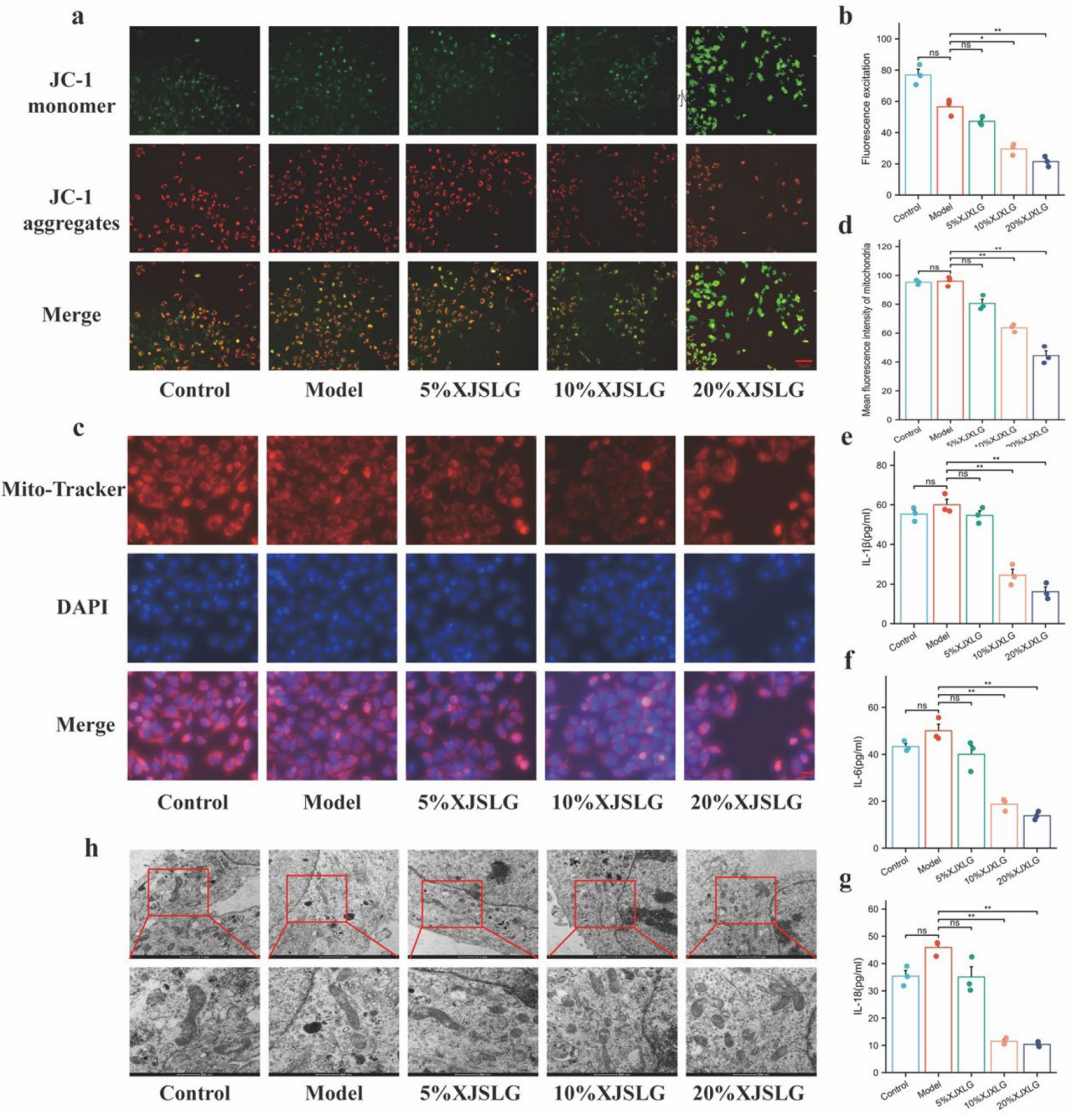
SJXLG improved mitochondrial function, morphology, and inflammatory cytokine levels in CORT-treated MCF-7 cells in vitro. **a** JC-1 assay of CORT-MCF-7 cells treated with SJXLG-containing serum (5%, 10%, 20%). **b** Quantification of JC-1. **c** MitoTracker assay of CORT-MCF-7 cells treated with SJXLG-containing serum (5%, 10%, 20%). **d** Quantification of MitoTracker. **e** IL-1 levels in each group. **f** IL-6 levels in each group. **g** IL-18 levels in each group. **h** Mitochondrial morphology observed by TEM in control and SJXLG-treated groups. Data are presented as mean ± SD,* n* = 3. *P < 0.05, **P < 0.01 versus control

Ex vivo analyses revealed elevated levels of IL-1β, IL-18, and IL-6 in the supernatants of the model group compared with controls, although differences were not statistically significant. However, SJXLG treatment reduced inflammatory cytokine levels in a dose-dependent manner, with significant decreases at 10% and 20% concentrations (*P* < 0.01, Fig. 4e–g). These data suggest that SJXLG exerts regulatory effects on inflammatory responses in the BCD ex vivo model.

### SJXLG ameliorates mitochondrial dysfunction and morphological alterations in CORT-treated MCF-7 cells

JC-1 staining showed that MCF-7 cells exhibited red aggregates under high mitochondrial membrane potential, whereas green monomers were observed under low mitochondrial membrane potential. CORT-treated MCF-7 cells in the model group displayed an increase in red monomers and a decrease in green aggregates. After SJXLG treatment, green fluorescence was markedly enhanced, and the mitochondrial membrane potential was significantly reduced (Fig. 4a,b), indicating that SJXLG exerts a disruptive effect on the mitochondrial membrane potential of MCF-7 cells.

MitoTracker staining and ImageJ-based analysis revealed distinct alterations in mitochondrial morphology. In the solvent control group, mitochondria displayed elongated, interconnected filamentous networks. Model group cells exhibited more intact mitochondria with increased numbers and branches. In contrast, SJXLG-treated cells showed decreased mitochondrial numbers, with smaller, rounded structures and fewer branches (Fig. 4c,d).

TEM further confirmed these findings, showing mitochondrial shrinkage, reduced cristae, and increased bilayer membrane density in SJXLG-treated model cells (Fig. 4h). Collectively, these results indicate that SJXLG induces structural damage to mitochondria in breast cancer cells.

### Bioinformatics analysis of SJXLG intervention in breast cancer–related depression

**Fig. 5:**
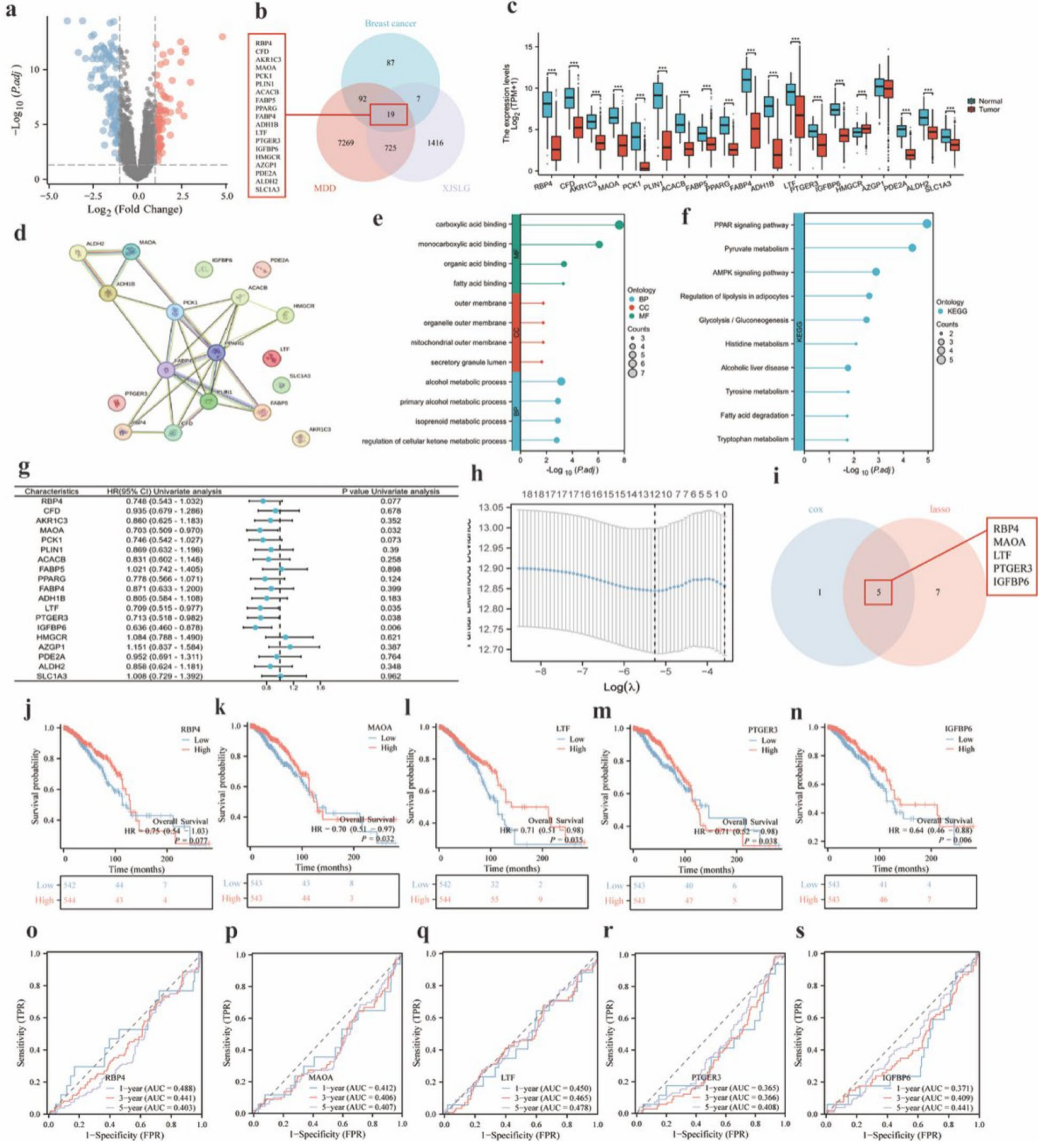
Bioinformatics analysis of SJXLG in breast cancer with depression. **a** Volcano plot of differentially expressed genes in GSE15852. **b** Venn diagram of SJXLG intervention targets, breast cancer DEGs, and MDD-related genes. **c** Validation of differential expression of SJXLG intervention targets related to depression-associated breast cancer. **d** PPI network of 19 key genes regulated by SJXLG in depression-related breast cancer. **e** GO enrichment analysis. **f** KEGG enrichment analysis. **g** Forest plot of Cox regression analysis of SJXLG-regulated depression-related breast cancer genes. **h** LASSO analysis of SJXLG-regulated depression-related breast cancer genes. **i** Intersection of prognostic genes from Cox and LASSO analyses. **j**–**n** Survival regression fitting of RBP4, MAOA, LTF, PTGER3, and IGFBP6. **o**–**s** Predictive performance analysis of RBP4, MAOA, LTF, PTGER3, and IGFBP6. Data are presented as mean ± SD,* n* = 3. *P < 0.05, **P < 0.01 versus control

Using the GEO database, a breast cancer–related dataset (GSE15852) was screened, and differential expression analysis identified 205 differentially expressed genes (Fig. 5a). Subsequently, 16,230 major depressive disorder–related genes were retrieved from the GeneCards database, of which 8,105 genes were retained following median filtering (Fig. 5b). Integrating these results with the SJXLG compound–target network, intersection analysis identified 19 potential therapeutic targets of SJXLG in depression-related breast cancer, including RBP4, CFD, AKR1C3, MAOA, PCK1, PLIN1, ACACB, FABP5, PPARG, FBP4, ADH1B, LTF, PTGER3, IGFBP6, HMGCR, AZGP1, PDE2A, ALDH2, and SLC1A3 (Fig. 5c,d).

Further GO and KEGG enrichment analyses revealed significant enrichment of pathways associated with lipid metabolism, inflammatory regulation, receptor-mediated signaling, and neurotransmitter-related processes, suggesting that SJXLG may exert critical modulatory effects in the pathogenesis and progression of depression-related breast cancer (Fig. 5e,f).

RNA-seq data from TCGA (https://portal.gdc.cancer.gov) were analyzed, revealing five SJXLG target genes (RBP4, MAOA, LTF, PTGER3, IGFBP6) that were closely associated with prognosis in breast cancer patients (Fig. 5g–i). Kaplan–Meier survival curves indicated that RBP4 expression was not significantly correlated with prognosis (*P* = 0.077 > 0.05), whereas high expression of MAOA, LTF, PTGER3, and IGFBP6 was significantly associated with favorable prognosis (*P* = 0.032, 0.035, 0.038, 0.006 < 0.05), and all four genes served as protective factors (HR < 1) (Fig. 5j–n).

In the timeROC analysis, the software package by default defines patients experiencing events at a given time point as the “case group.” Consequently, when the studied indicators act as protective factors (HR < 1), the AUC value often appears < 0.5. In such instances, a smaller AUC value closer to 0 paradoxically reflects stronger prognostic performance, although the prediction direction is opposite to the predefined event definition. Therefore, interpretation of clinical significance should be integrated with the results of Cox regression analysis. The timeROC curve results demonstrated that MAOA, LTF, PTGER3, and IGFBP6 possess prognostic value and act as protective factors in breast cancer patients (AUC < 0.5), among which PTGER3 exhibited the most favorable prognostic efficacy (1-year AUC = 0.365; 3-year AUC = 0.366; 5-year AUC = 0.408) (Fig. 5o–s).

Importantly, functional annotation of these prognostically relevant targets revealed close involvement in lipid metabolism (MAOA, IGFBP6), inflammatory and immune regulation (LTF), and prostaglandin-mediated receptor signaling (PTGER3). These biological processes showed substantial overlap with the metabolic pathways identified in the untargeted metabolomics analysis (Sect. 3.9), indicating a potential molecular–metabolic regulatory axis underlying the therapeutic effects of SJXLG.

### Effects of SJXLG on depressive-like behaviors in BCD model mice

**Fig. 6:**
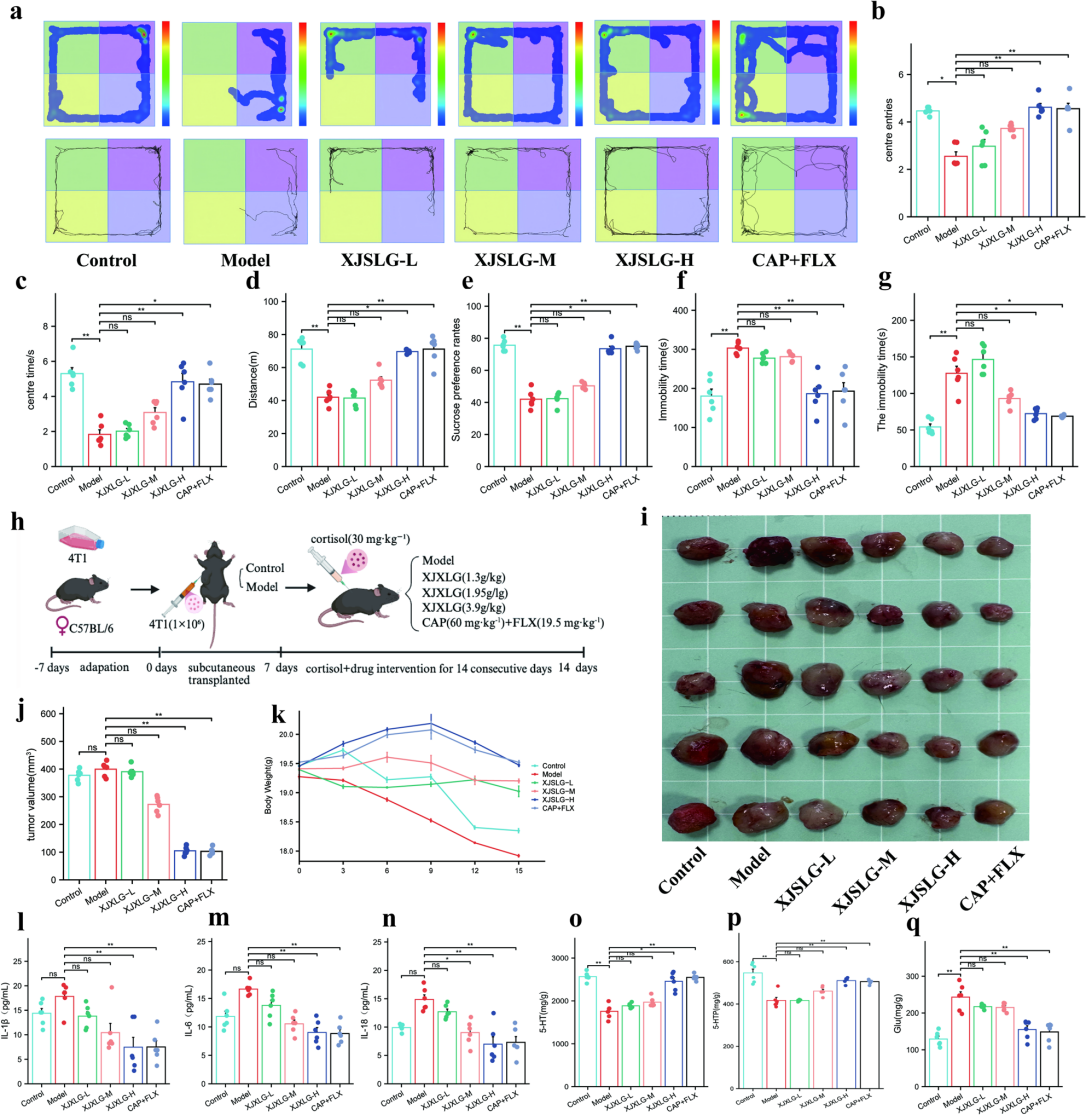
SJXLG suppressed depressive-like behaviors and tumor growth in a breast cancer depression model in vivo. **a** Representative locomotor trajectories and heat maps in the OFT. **b** Entries into the center zone in OFT. **c** Time spent in the center zone. **d** Distance traveled in the center zone. **e** Results of sucrose preference test. **f** Immobility time in the tail suspension test. **g** Immobility time in the forced swim test. **h** Timeline of tumor model establishment and drug intervention in C57BL/6 mice. **i** Tumor volume analysis. **j** Body weight monitoring. **k** Representative images of excised tumors. **l**–**n** IL-1, IL-6, and IL-18 levels in each group. **o**–**q** Levels of 5-HTP, 5-HT, and Glu in each group. Data are presented as mean ± SD,* n* = 5. *P < 0.05, **P < 0.01 versus control

A series of behavioral tests, including the sucrose preference test, open field test, tail suspension test, and forced swim test, demonstrated that SJXLG effectively alleviated depressive-like symptoms in BCD mice. In the open field test, compared with the control group, the model group exhibited significantly reduced total locomotor distance and duration, whereas mice treated with SJXLG-H showed markedly increased activity, indicating that SJXLG mitigated fatigue and loss of vitality associated with breast cancer–related depression (Fig. 6a–d).

In the sucrose preference test, sucrose consumption was significantly reduced in the model group, while SJXLG treatment restored sucrose intake, with SJXLG-H producing statistically significant effects, further confirming its preventive role against BCD (Fig. 6e).

In both the tail suspension test and the forced swim test, immobility times were significantly lower in SJXLG groups compared with the model group, with SJXLG-H achieving statistical significance. These findings suggest that SJXLG effectively alleviates depressive-like behaviors in BCD mice (Fig. 6f–g).

### SJXLG inhibits tumor growth in the BCD mouse model

**Fig. 7:**
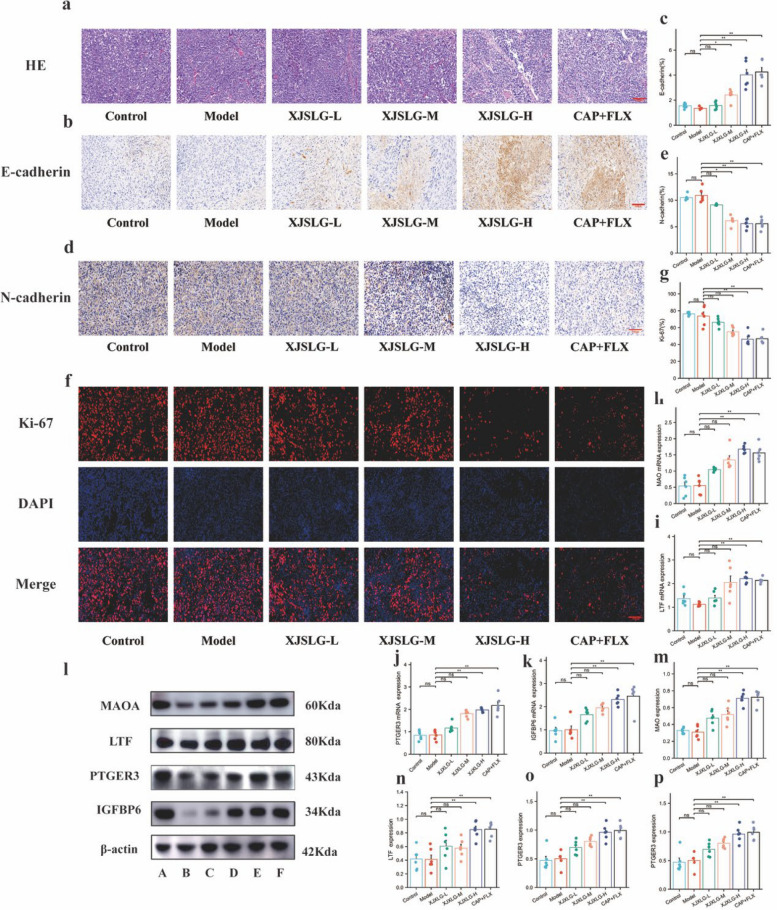
SJXLG suppressed tumor cell proliferation in vivo and explored underlying mechanisms in the breast cancer depression model. **a** HE staining of tumor tissues. **b** Immunohistochemical staining of E-cadherin. **c** Quantification of E-cadherin-positive expression. **d** Immunohistochemical staining of N-cadherin. **e** Quantification of N-cadherin-positive expression. **f** Immunofluorescence staining of Ki-67. **g** Quantification of Ki-67-positive cells. **h**–**k** mRNA expression of MAOA, LTF, PTGER3, and IGFBP6. **l** Western blot analysis of LTF, MAOA, PTGER3, and IGFBP6. **m**–**p** Densitometric analysis of MAOA, LTF, PTGER3, and IGFBP6. Data are presented as mean ± SD, *n* = 5. *P < 0.05, **P < 0.01 versus control

In a 4T1 breast cancer xenograft model established in C57BL/6 mice (Fig. 6h), both SJXLG-H and capecitabine plus fluoxetine treatment significantly inhibited tumor growth, as evidenced by reduced tumor volume (*P* < 0.01) (Fig. 6i–k). Histopathological analysis using H&E staining revealed densely packed tumor tissues with marked hyperproliferation and well-defined cell boundaries in the control and model groups. In contrast, SJXLG and capecitabine plus fluoxetine groups displayed extensive necrosis characterized by karyopyknosis and karyorrhexis, suggesting reduced tumor cell density and viability (Fig. 7a).Immunofluorescence staining showed that Ki-67 expression, a marker of cell proliferation, was significantly decreased following SJXLG-H and capecitabine plus fluoxetine treatment (*P* < 0.01) (Fig. 7f–g).Immunohistochemical analysis further revealed increased E-cadherin and decreased N-cadherin expression in the SJXLG and capecitabine plus fluoxetine groups, indicating that SJXLG significantly suppressed breast cancer cell proliferation compared with the model group (*P* < 0.05,* P* < 0.01) (Fig. 7b–e).

### Biomarker analysis in BCD mice following SJXLG treatment

To evaluate the therapeutic potential of SJXLG in BCD mice, serum biomarkers were assessed. In the model group, levels of IL-1β, IL-18, and IL-6 were significantly elevated, reflecting a systemic inflammatory response induced by the combined effects of breast cancer and depression. Treatment with SJXLG or capecitabine plus fluoxetine markedly reversed these elevations, restoring cytokine levels toward normal (*P* < 0.05, *P* < 0.01) (Fig. 6l–m).

Regarding neuroendocrine function, serum 5-HTP and 5-HT levels were significantly reduced in the model group, whereas SJXLG-H and capecitabine plus fluoxetine significantly increased both 5-HTP and 5-HT levels while reducing Glu levels. These findings suggest that SJXLG exerts beneficial effects by modulating neurotransmitter balance and attenuating inflammatory responses (*P* < 0.05, *P* < 0.01) (Fig. 6o–q).

### Mechanistic investigation of SJXLG in suppressing BCD tumor growth in vivo

To further elucidate the mechanisms underlying the inhibitory effects of SJXLG on tumor growth in the BCD mouse model, tumor tissues from treated animals were subjected to qRT-PCR and WB analysis. The expression levels of key genes and proteins, including MAOA, LTF, PTGER3, and IGFBP6, were assessed. The results demonstrated that both SJXLG and the combination of capecitabine plus fluoxetine significantly upregulated the protein and mRNA expression of MAOA, LTF, PTGER3, and IGFBP6 in tumor tissues (*P* < 0.05, *P* < 0.01) (Fig. 7h–p). By contrast, SJXLG-L failed to elicit a significant effect on these targets (*P* > 0.05). These findings suggest that the antiproliferative effects of SJXLG in BCD mice may be mediated through the suppression of MAOA, LTF, PTGER3, and IGFBP6, which are implicated in tumor regulation.

### Untargeted metabolomics identifies lipid and amino acid metabolic reprogramming mediated by SJXLG in breast cancer with comorbid depression

**Fig. 8:**
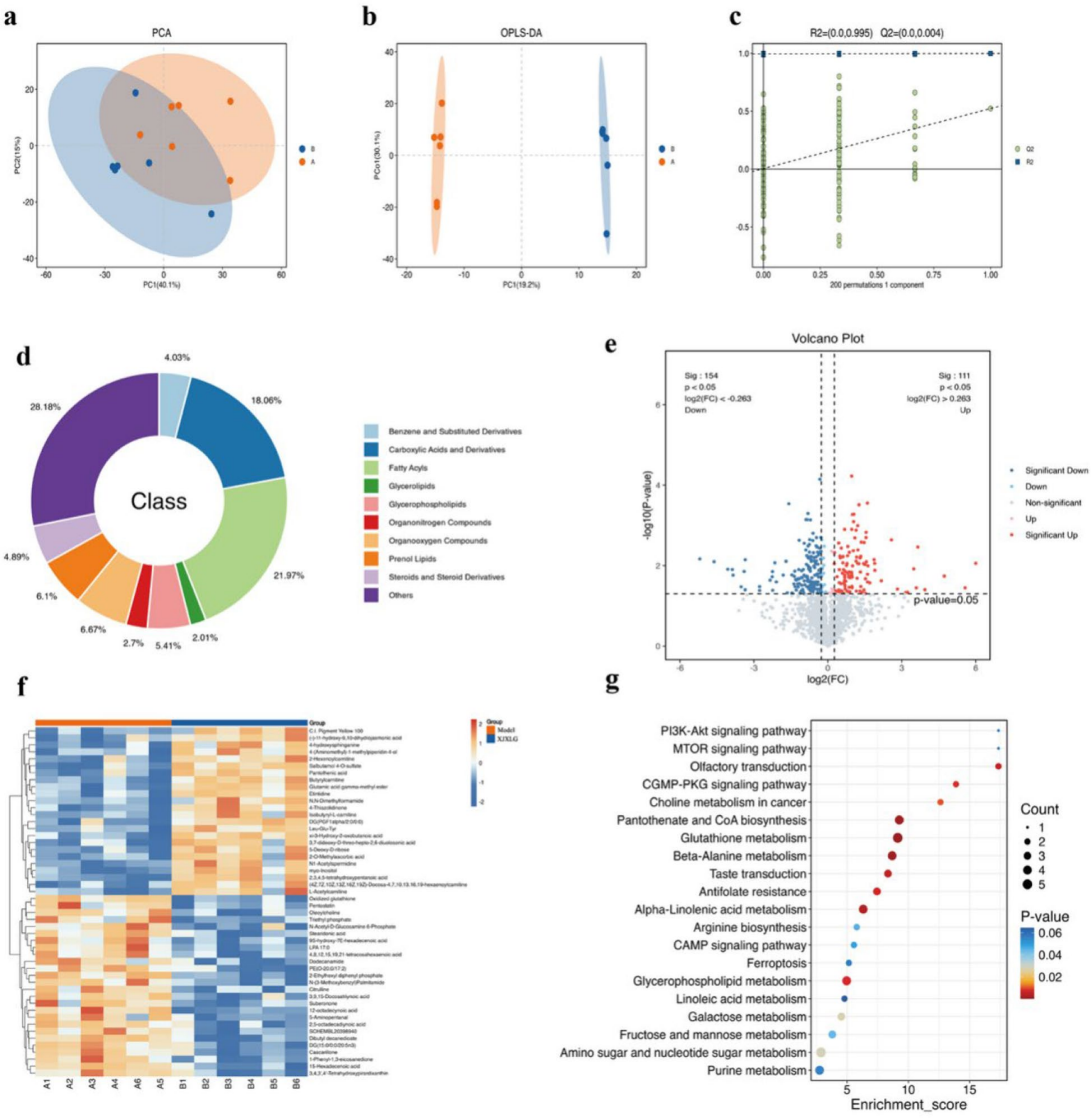
Untargeted metabolomic analysis of tumor tissues following SJXLG treatment. **a** Principal component analysis (PCA) illustrated a clear distinction in metabolic profiles between the control and AA-treated groups. **b** Orthogonal partial least squares discriminant analysis (OPLS-DA) score plots further highlighted well-separated clustering of the two groups. **c** The OPLS-DA permutation test verified the stability and predictive reliability of the model, with R^2^Y = 0.995 and Q^2^ = 0.004 after 200 permutations, indicating strong model fitness without signs of overfitting. **d** Chemical taxonomy classification revealed that fatty acyls represented the largest category among differential metabolites. **e** The volcano plot visualized significantly altered metabolites based on the thresholds VIP > 1, |log₂FC|> 0.263, and *p* < 0.05. **f** The heatmap of the top 50 metabolites displayed distinct abundance patterns across samples, with most changes associated with lipid metabolism and membrane signaling regulation. **g** KEGG pathway enrichment analysis identified the major metabolic pathways affected by SJXLG intervention

To investigate the metabolic regulatory effects of SJXLG on breast cancer with comorbid depression, untargeted metabolomics analysis was performed on tumor tissues from the model and SJXLG-treated groups.PCA revealed a clear separation trend between the two groups, while OPLS-DA further demonstrated significant metabolic differences, with R^2^Y = 0.995 and Q^2^ = 0.004 (Fig. 8a–c), indicating the model’s strong stability and predictive reliability.

Chemical classification of the differential metabolites showed that fatty acyls accounted for the largest proportion (21.97%), suggesting a key role of lipid metabolism in the model group. Carboxylic acids and their derivatives represented 18.06%, reflecting active amino acid and small-molecule organic acid metabolism, while steroids and steroid derivatives accounted for 4.89%, indicating potential involvement in hormonal metabolism. In total, 244 differential metabolites were identified (VIP > 1.0, FC > 1.5 or < 0.67, *p* < 0.05), including 106 upregulated and 138 downregulated compounds (Fig. 8 d–e).

In the model group, metabolites associated with lipid metabolism and membrane signaling—such as 4-hydroxysphinganine, isobutyryl-L-carnitine, stearidonic acid, and oleoylcholine—were significantly elevated but markedly decreased after SJXLG treatment. This suggests that the formula may modulate sphingolipid and acylcholine metabolism, thereby influencing membrane signaling and inflammatory responses. Moreover, LPA 17:0 and DG(15:0/0:0/20:5n3), which were upregulated in the model group, returned to normal levels following treatment, implying their involvement in inflammatory signaling and cell proliferation processes.

KEGG pathway enrichment analysis indicated that SJXLG primarily affected signal transduction and metabolic regulation pathways, including the PI3K-Akt, mTOR, and cGMP-PKG signaling pathways, which are closely related to cell proliferation, survival, and neurotransmitter signaling. In addition, pathways related to energy, lipid, and amino acid metabolism, such as choline metabolism in cancer, glutathione metabolism, beta-alanine metabolism, arginine biosynthesis, linoleic acid metabolism, and fructose and mannose metabolism, were significantly enriched.

To further integrate metabolomic and bioinformatic findings, Spearman correlation analysis was performed between significantly altered metabolites and the expression levels of key prognostic targets identified in Sect. 3.4 (MAOA, LTF, PTGER3, and IGFBP6). The results revealed that multiple lipid- and amino acid–related metabolites showed significant correlations with these targets. Notably, sphingolipid- and acylcarnitine-associated metabolites exhibited strong correlations with MAOA and IGFBP6, while inflammatory lipid mediators were closely associated with LTF and PTGER3 expression. These associations suggest that SJXLG may regulate tumor growth and depressive behaviors through coordinated modulation of metabolic reprogramming and target gene expression.

In summary, SJXLG may exert integrated anti-tumor and antidepressant effects by coordinately regulating lipid, amino acid, and energy metabolic networks, improving the tumor microenvironment, alleviating oxidative stress, and modulating neurotransmitter metabolism (Fig. 8 f–g). The correlation between key metabolites and prognostically relevant targets provides mechanistic evidence supporting a molecular–metabolic regulatory axis underlying the therapeutic efficacy of SJXLG in breast cancer with comorbid depression.

## Discussion

Breast cancer is one of the most prevalent malignancies among women worldwide, with steadily rising incidence and mortality rates, posing a substantial threat to women’s health and socioeconomic stability. Although advances in therapeutic strategies have improved overall survival, long-term treatment is frequently accompanied by significant psychological comorbidities, most notably depression [22]. Epidemiological data indicate that the prevalence of depression among breast cancer patients is considerably higher than in the general population, with some studies reporting rates as high as 25–30% [23]. The etiology of this comorbidity is multifactorial, encompassing body image concerns following mastectomy, fear of recurrence, treatment-related side effects, and inadequate social support. Importantly, depression not only undermines quality of life and treatment adherence but may also promote tumor initiation and progression through neuroendocrine, immune-inflammatory, and metabolic pathways, thereby creating a vicious cycle [24].

Current therapeutic approaches for breast cancer and depression remain largely segregated, with anticancer treatment and psychological management conducted independently [25]. Conventional anticancer regimens, such as endocrine therapy, targeted therapy, and chemotherapy, while effective in prolonging survival, are limited by drug resistance, adverse effects, and single-target specificity [26]. For instance, endocrine therapy predominantly targets hormone-dependent signaling, whereas chemotherapy mainly suppresses rapidly proliferating cells, often neglecting inflammation-, stress-, and metabolism-related mechanisms that are closely associated with depressive symptoms. Likewise, antidepressants such as selective serotonin reuptake inhibitors (SSRIs) can alleviate mood symptoms but raise concerns regarding drug–drug interactions, altered cytochrome P450 activity, and potential modulation of tumor immune surveillance in oncological contexts [27]. Moreover, most antidepressants act primarily on monoaminergic neurotransmission and do not address tumor-associated metabolic reprogramming or inflammatory dysregulation, highlighting the limitations of single-target pharmacotherapy in BCD.

Traditional Chinese Medicine (TCM), emphasizing the holistic regulation of both “body and mind,” has accumulated extensive clinical experience in managing complex pathological states [13]. In contrast to conventional mono-target agents, multi-herbal formulations are characterized by network-based pharmacological actions, enabling simultaneous modulation of tumor growth, immune inflammation, metabolic balance, and emotional regulation [28]. Guided by the TCM principle of “replenishing Qi, harmonizing emotions, and resolving stagnation,” SJXLG has long been applied in clinical practice for tumors accompanied by emotional disorders.

This study systematically evaluated the effects of SJXLG in breast cancer complicated with depression and explored its mechanisms through phytochemical profiling, in vitro and in vivo experiments, and bioinformatics analyses. LC–MS identified 24 active compounds, several of which—including baicalin, ursolic acid, and saikosaponin—have been previously validated for their antitumor, anti-inflammatory, antioxidant, and neuromodulatory activities. Unlike single-molecule antidepressants or chemotherapeutic agents, these constituents are likely to act synergistically, forming a multi-layered regulatory network that may reduce compensatory pathway activation and therapeutic resistance.

In vitro assays revealed that SJXLG significantly inhibited the proliferation, migration, and invasion of breast cancer cells in a dose-dependent manner. In vivo, SJXLG markedly reduced tumor volume, decreased serum levels of inflammatory cytokines (IL-1β, IL-18, and IL-6), and ameliorated depression-like behaviors in mice, such as reduced sucrose preference and prolonged immobility in the forced swim test. Notably, compared with standard antidepressants that primarily improve behavioral outcomes without influencing tumor biology, SJXLG simultaneously alleviated depressive-like behaviors and suppressed tumor progression, underscoring its integrative therapeutic potential in BCD.

In terms of signaling pathways, bioinformatics analysis revealed that SJXLG could regulate multiple pathways, including the PPAR signaling pathway, AMPK signaling pathway, lipid metabolism, and neurotransmitter synthesis. These pathways are rarely co-targeted by existing anticancer or antidepressant drugs, which typically act on isolated signaling axes. In addition, untargeted metabolomics analysis indicated that SJXLG may exert its therapeutic effects through coordinated regulation of lipid, amino acid, and energy metabolic networks, thereby alleviating oxidative stress and restoring neurotransmitter balance. These pathways collectively play crucial roles in modulating metabolic status, cellular energy homeostasis, immune-inflammatory responses, and neural regulation in breast cancer [29, 30].

Moreover, four key genes—MAOA, LTF, PTGER3, and IGFBP6—were identified as potential targets closely associated with both breast cancer prognosis and emotional regulation. Unlike conventional agents that modulate a single molecular target (e.g., MAOA inhibitors in depression), SJXLG appears to fine-tune multiple interconnected targets involved in neurotransmitter metabolism, inflammation, immune regulation, and growth factor signaling, potentially reducing the risk of excessive pathway inhibition and adverse effects. Excessive MAOA activation is known to increase ROS generation; accordingly, SJXLG-mediated regulation of MAOA may contribute to reduced oxidative stress and lipid peroxidation [31]. LTF, PTGER3, and IGFBP6 further participate in inflammatory modulation, neuroimmune signaling, and PI3K–Akt/mTOR-mediated metabolic regulation, consistent with the metabolomic enrichment of inflammation- and lipid-related pathways [32, 33].

Pathophysiological links between inflammation, mitochondrial dysfunction, and tumor–depression comorbidity were also evident. Proinflammatory cytokines triggered by mitochondrial ROS and mtDNA release activate inflammasomes and amplify IL-1β/IL-18 and IL-6 signaling cascades [34]. Importantly, conventional chemotherapeutic agents may further exacerbate mitochondrial damage and oxidative stress, potentially aggravating fatigue and depressive symptoms. In contrast, SJXLG appears to mitigate this vicious cycle by modulating inflammatory signaling, oxidative stress, and mitochondrial-associated metabolic pathways [35, 36].

**Fig. 9:**
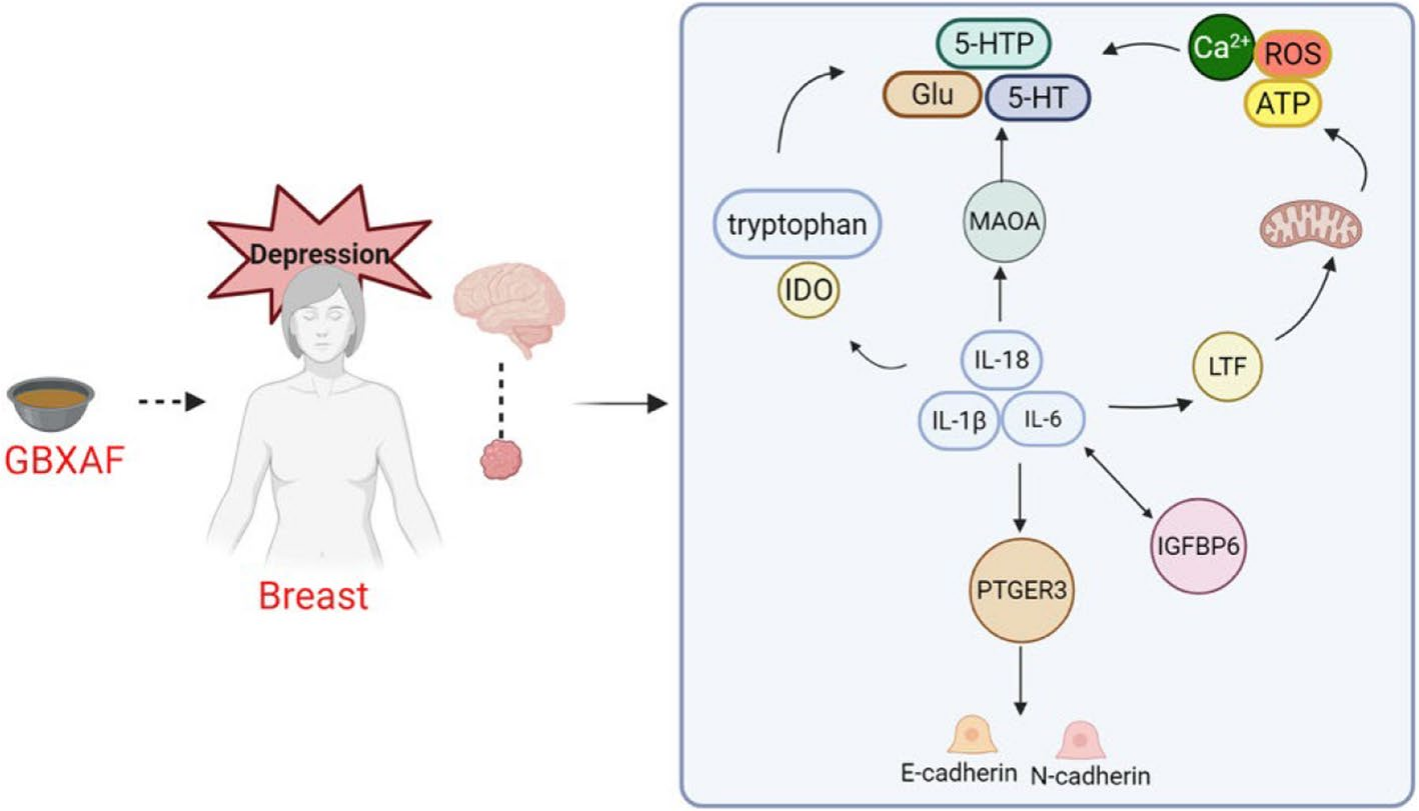
Proposed schematic diagram of the mechanisms underlying the effects of SJXLG on breast cancer with depression

From a safety perspective, SJXLG demonstrated no observable systemic toxicity in vivo, contrasting with the well-documented adverse profiles of chemotherapy and some antidepressants. Its multi-target yet moderate regulatory pattern may allow effective intervention without excessive pathway blockade, potentially improving treatment tolerance, adherence, and long-term quality of life in patients with BCD (Fig. 9). Nevertheless, further functional validation and clinical studies are required to confirm causality and translational relevance.

In summary, this study reveals the multi-target, synergistic actions of SJXLG against breast cancer and depression through molecular, cellular, behavioral, and systems-level evidence. Compared with existing single-target anticancer and antidepressant therapies, SJXLG offers an integrative, network-based therapeutic strategy, providing a scientific basis for the ethnopharmacological application of multi-herbal prescriptions in complex tumor–psychiatric comorbidities.

## Conclusion

In summary, the present study provides comprehensive evidence that SJXLG exerts dual anticancer and antidepressant effects in breast cancer with depression (BCD) comorbidity through multi-target and multi-pathway regulation. By integrating pharmacological, bioinformatic, and metabolomic analyses, SJXLG was shown to suppress tumor growth, modulate inflammatory and neuroendocrine responses, restore metabolic homeostasis, and alleviate depression-like behaviors. These findings suggest that SJXLG achieves synergistic regulation of the tumor microenvironment and emotional balance, thereby demonstrating a distinctive advantage in holistic intervention. Collectively, this study highlights SJXLG as a promising multi-component herbal formulation for managing complex comorbidities involving both physiological and psychological dysfunctions. Future studies should focus on elucidating the contribution of individual active constituents, pharmacokinetic characteristics, and molecular targets, and further extend these findings into well-designed clinical validations.

## Data Availability

No datasets were generated or analysed during the current study.

## Abbreviations

BCD: Breast Cancer with Depression
BC: Breast Cancer
TCM: Traditional Chinese Medicine
SJXLG: Xiaojie Sanliu Granules
MEM: Minimum Essential Medium
DMEM: Dulbecco’s Modified Eagle Medium
FBS: Fetal Bovine Serum
CORT: Corticosterone
CCK-8: Cell Counting Kit-8
EdU: 5-Ethynyl-2´-deoxyuridine
JC-1: 5,5’,6,6’-Tetrachloro-1,1’,3,3’-tetraethylbenzimidazolylcarbocyanine iodide
TEM: Transmission Electron Microscopy
ELISA: Enzyme-Linked Immunosorbent Assay
OFT: Open Field Test
TST: Tail Suspension Test
SPT: Sucrose Preference Test
FST: Forced Swim Test
H&E: Hematoxylin & Eosin staining
IHC: Immunohistochemistry
IF: Immunofluorescence
qRT-PCR: Quantitative Real-Time Polymerase Chain Reaction
WB: Western Blotting
GAPDH: Glyceraldehyde-3-Phosphate Dehydrogenase
PPI: Protein–Protein Interaction
GO: Gene Ontology
KEGG: Kyoto Encyclopedia of Genes and Genomes
TCMSP: Traditional Chinese Medicine Systems Pharmacology Database
TCGA: The Cancer Genome Atlas
GEO: Gene Expression Omnibus
OB: Oral Bioavailability
DL: Drug-Likeness
LC–MS: Liquid Chromatography–Mass Spectrometry
HESI: Heated Electrospray Ionization
SDS-PAGE: Sodium Dodecyl Sulfate Polyacrylamide Gel Electrophoresis
LSD: Least Significant Difference
